# Congenital giant epulis obstructing oral cavity: newborn emergency

**DOI:** 10.11604/pamj.2014.17.195.3023

**Published:** 2014-03-13

**Authors:** Komla Gnassingbe, Komlan A Mihluedo-Agbolan, Harefetéguéna Bissa, Koffi Amegbor, Nguefack Blanchard Noumedem, Pilakimwe Egbohou, Wakatou Mama, Gamedzi K Akakpo-Numado, Hubert Tekou

**Affiliations:** 1 Pediatric Surgery Department Sylvanus Olympio Teaching Hospital, Lomé, Togo; 2 Stomato- ondontology department, Sylvanus Olympio Teaching Hospital, Lomé, Togo; 3 Anatomo pathology department, Sylvanus Olympio Teaching Hospital, Lomé-Togo; 4 Emergency and Anesthesia Department, Sylvanus Olympio Teaching Hospital, Lomé, Togo

**Keywords:** Congenital epulis, newborn, oral cavity, Togo

## Abstract

The congenital epulis is a benign congenital granular cell tumor arising most often of the alveolar ridge of the jawbone. When giant, it is source of digestive discomfort disabling feeding. We report the case of a newborn female, vaginal delivery, presented with a giant intraoral tumor. Tumor obstructing the mouth of the newborn and prevent the attachment and feeding. The treatment consisted of excision of the tumor under general anesthesia. The histology of the tumor was revealed that it was an epulis.

## Introduction

The congenital epulis also called Neumann tumor or congenital granular cell tumor is a benign tumor of the oropharyngeal child. This is a rare disease [1–4], the first description was made by Neumann in 1971 [1, 2]. Since the first description, less than 200 publications have been made on this disease around the world [1]. It presents as a single or multiple sessile or pedunculated tumor, arising most often of the alveolar ridge of the jawbone. When giant, it is causing a respiratory and digestive discomfort especially in preventing feeding. The aetiology of epulis is still unknown [1, 4]. The diagnosis of epulis is clinical and confirmed by pathological examination of the biopsy specimen excision. Its treatment ranges from simple monitoring to surgical excision when the tumor is giant. The prognosis of this condition is usually favorable without recurrence [4]. We report a unique case of congenital epulis giant in granulosa cells in a newborn in order to do a review of the literature on the clinicopathological features, scalable and therapeutic of this rare entity.

## Patient and observation

**Figure 1:**
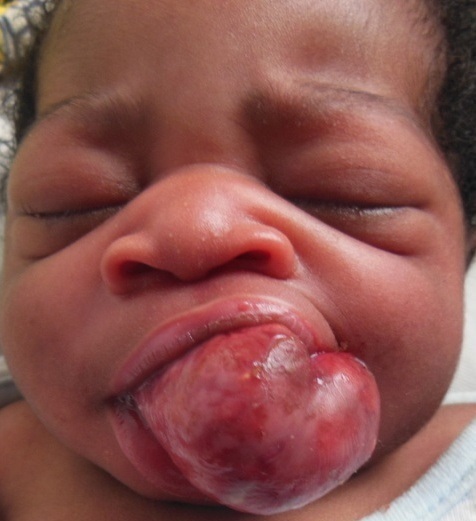
Appearance of the tumor in the oral mucosa

**Figure 2:**
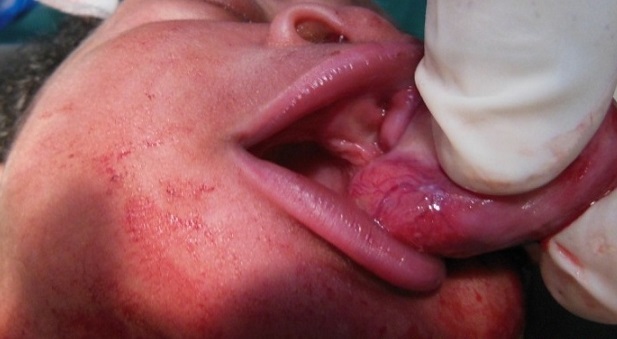
Shape showing the location of the tumor (sessile tumor)

**Figure 3:**
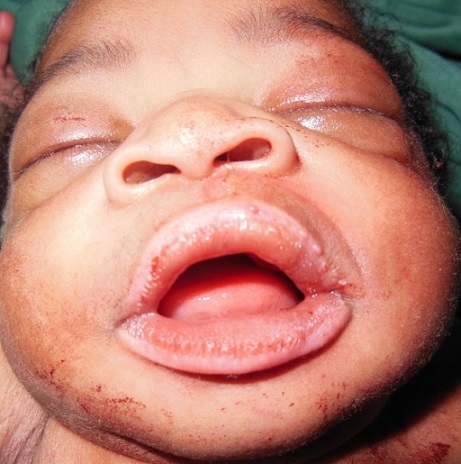
Immediate postoperative appearance (after removal of the tumor)

**Figure 4:**
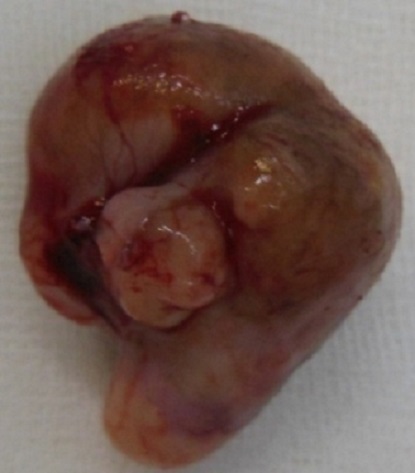
Appearance of the tumor after its removal

**Figure 5:**
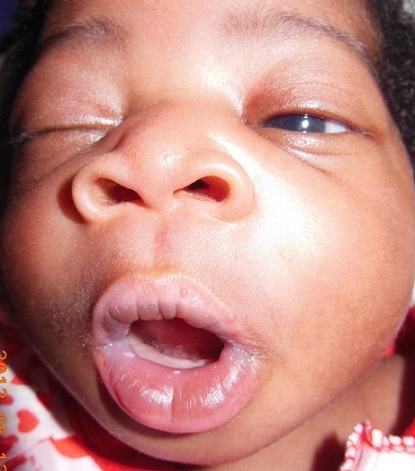
Appearance two weeks after removal of the tumor

Newborn female on the first day of extra uterine life referred in March 8th, 2013 from the maternity of Sylvanus Olympio Teaching Hospital to surgical emergencies of the same hospital for intraoral tumor. It was after a full-term pregnancy (37 weeks of amenorrhea), during which, no related-pregnancy pathology nor unrelated ones was discovered. There was no concept of taking foetal toxic drug by the mother. Obstetric ultrasonography at the 12th week of gestation had objectified no malformations. The delivery was done vaginally without incident in March 8, 2013 with an Apgar score of 10-10-10 and a weight of 3050 grams. Examination of the newborn at her admission noted an intraoral tumor pink, firm, pedunculated sitting in the gingival surface of upper left of the midline cleft (Figure 1, Figure 2) maxilla. The tumor measured 6 centimeters long axis and obstructed the entire oral cavity, thus preventing the taking of the womb and feed. The rest of the intraoral mucosa was normal. The newborn showed no respiratory disorder. There were no other clinically visible malformations. A peripheral vein was taken for an infusion of hypertonic glucose solution 10% to fight against possible hypoglycaemia. Surgical treatment was performed and consisted of tumor resection under general anesthesia. Figure 3 shows the immediate postoperative appearance. Histologically, the tumor was formed by a proliferation of tumor large, round or fusiform with abundant cytoplasm, and eosinophilic granular cells with a round nucleus. On the surface, the tumor was covered by squamous epithelium regular kind. Figure 4 shows the appearance of the tumor after its removal. Control of the child two weeks after surgery showed a healthy intraoral mucosa without tumor recurrence (Figure 5).

## Discussion

The giant congenital epulis is a rare disease [4–6]. In fact, less than 200 publications have been made on this disease in the world [1, 4]. This is the first of a typical case in the Pediatric Surgery department of the Sylvanus Olympio teaching hospital (Lomé). It is the prerogative of newborns as shown by our observation and mainly affects girls [1, 4, 6]. Aetiology is not yet known for this condition but hormonal factors have been suspected during pregnancy [1, 7]. In our case, no aetiology was found.

The tumor is localized most often at the expense of the mucosa of the maxillary alveolar ridge [4, 8] as found in our observation. Diagnosis is mainly clinical postnatal by the discovery of an intraoral mass. However, the discovery can be made by prenatal obstetric ultrasound from the 36th week of gestation [4, 8, 9]. Prenatal diagnosis remains difficult in our country due to the inaccessibility of the review by the women and the lack of experienced in ultrasound in antenatal diagnosis. To all this is added the absence of modern ultrasound device. In our case, the tumor size was six centimeters and obstructing the oral cavity and preventing breast feeding. According to Chaari et al [4], as Wittebole et al [10], the size of epulis up to nine centimeters and in some cases causing respiratory and digestive gene.

The congenital epulis clinically pose a diagnosis problem with leiomyoma, congenital dermoid cyst, congenital choristoma, congenital fibrosarcoma, lipoma and congenital fibrous epulis [4, 11]. The differential diagnosis is made by histological examination reveals that in the case of congenital epulis proliferation in regular layers of granulosa cells positive staining Periodic Acid Schiff (PAS). These cells are grown in a plexiform vascular stroma characteristic lifting surface regularly mucosa [1, 4, 6, 12]. The same results were found in this case except that the PAS staining as well as immunohistochemical studies have not been carried out because of the limitation of our technical laboratory of Pathology of the Sylvanus Olympio Teaching Hospital.

We conducted a surgical excision of the tumor under general anesthesia. The same therapeutic approach has been found by several authors [1, 4, 6, 12]. However, in the publication of Barbuta et al. [13], cases of spontaneous regression of the tumor have been described. The outcome after surgical removal is generally favorable. No cases of recurrence, damage to future dentition [12], malignant transformation, distant metastases have been reported [8]. In our case our retreat was two weeks without recurrence.

## Conclusion

The congenital epulis is a rare benign intraoral tumor that often affects newborn female. Their aetiology is still unknown, but many authors criminalizes hormonal (oestrogen and progesterone) maternal-foetal. The diagnosis is clinical postnatal by the discovery of an intraoral tumor sitting on the maxillary alveolar mucosa. The confirmation of diagnosis is sent to histology. Treatment is usually surgical and prognosis is often good.
